# LncRNA USP2-AS1 Promotes Hepatocellular Carcinoma Growth by Enhancing YBX1-Mediated HIF1α Protein Translation Under Hypoxia

**DOI:** 10.3389/fonc.2022.882372

**Published:** 2022-05-25

**Authors:** Shi-Ping Chen, Gui-Qi Zhu, Xiao-Xia Xing, Jing-Lei Wan, Jia-Liang Cai, Jun-Xian Du, Li-Na Song, Zhi Dai, Jian Zhou

**Affiliations:** ^1^ Department of Liver Surgery and Transplantation, Liver Cancer Institute, Zhongshan Hospital, Fudan University; Key Laboratory of Carcinogenesis and Cancer Invasion of Ministry of Education, Shanghai, China; ^2^ Liver Cancer Institute, Zhongshan Hospital, Fudan University, Shanghai, China; ^3^ State Key Laboratory of Genetic Engineering, Fudan University, Shanghai, China; ^4^ Department of General Surgery, Zhongshan Hospital, Fudan University, Shanghai, China

**Keywords:** TME: tumor microenvironment, USP2-AS1: ubiquitin specific peptidase 2 antisense RNA 1, YBX1: Y-box binding protein 1, lncRNA, HIF1α, Lenvatinib, hypoxia, HCC

## Abstract

Recently, the role of lncRNAs in tumorigenesis and development has received increasing attention, but the mechanism underlying lncRNAs-mediated tumor growth in the hypoxic microenvironment of solid tumors remains obscure. Using RNA sequencing, 25 hypoxia-related lncRNAs were found to be upregulated in HCC, of which lncRNA USP2-AS1 were significantly increased under hypoxia. We further confirmed that USP2-AS1 was significantly upregulated in liver cancer using FISH assay and that USP2-AS1 was associated with advanced liver cancer and increased tumor size. Furthermore, overexpression of USP2-AS1 under hypoxia dramatically increased HCC proliferation and clone formation, whereas the opposite results were observed after USP2-AS1 knockdown. We also found that overexpression of USP2-AS1 increased migration and invasion of HCC cells, while USP2-AS1 knockdown led to the opposite effect. In addition, USP2-AS1 knockdown can increase the efficacy of lenvatinib in our mice tumor xenograft model. Our findings also suggest that USP2-AS1 could increase the protein level of HIF1α by enhancing YBX1 protein binding to HIF1α mRNA under hypoxia and the therapeutic effect of lenvatinib can be enhanced by combination with HIF1α inhibitors in liver cancer.

## Introduction

Liver cancer, including hepatocellular carcinoma (HCC) (80% of cases), is the fourth most common cancer and one of the leading causes of cancer-related mortality. About 50% of all liver cancer cases and deaths worldwide occur in China (1). HCC can be treated by different treatments, including liver transplantation, surgical resection, chemotherapy and radiation, targeted therapy, and immunotherapy. However, drug resistance and recurrence are common. Liver cancer has a five-year survival of less than 30% (2, 3). Therefore, insights into hepatocarcinogenesis are urgently needed to find effective therapeutic targets for HCC.

Hypoxia is one of the important features of the tumor microenvironment (TME) and is essential for tumor growth and progression (4). Hypoxia affects tumor angiogenesis (5), metabolic reprogramming (6), enhancing tumor invasion and metastasis ability (7), and immune escape (8, 9) by regulating different genes. Hypoxia-induced resistance to drug therapy has attracted a lot of attention from researchers (10–13). Hypoxia-inducible factor-1α (HIF1α) is a master regulator and interacts with upstream binding sites (called HIF regulatory elements) of hypoxia-responsive genes. Increased level of HIF1α has been shown to contribute to resistance to sorafenib and lenvatinib in HCC since antiangiogenic drug treatment results in insufficient oxygen supply, and elevated HIF1α and mediate the hypoxic adaptation of tumor cells (13, 14). Hypoxia-induced sorafenib resistance has been extensively investigated and METTL3 (methytrans ferase-like 3), HSP90α (heat shock protein 90 alpha), ATAD3A (ATPase family AAA domain-containing 3A), FOXO3A (forkhead box O3), have all been found to be involved in hypoxia-associated therapy resistance (15–18). However, the association of hypoxia-mediated abnormal lncRNAs expression and lenvatinib resistance remains uninvestigated.

Long non-coding RNAs (LncRNAs) are defined as non-coding RNA molecules that are longer than 200 nucleotides in length with limited or no protein-coding capacity and they have been shown to play significant roles in cancer (19, 20). Abnormal expression of lncRNAs mediates carcinogenesis, tumor progression, and invasion. Olivero et al. demonstrated that p53 activates the lncRNA Pvt1b to inhibit Myc and suppress tumorigenesis (21). SATB2-AS1 inhibits tumor metastasis by regulating SATB2 (22). LncRNAs also affect immunotherapeutic and chemotherapy efficacy and have different clinical and prognostic implications (23–26). However, the effect of hypoxia on the expression of lncRNAs remains elusive.

In this study, we identified 25 hypoxia-associated lncRNAs by RNA sequencing (RNA-seq) that are both upregulated in HCC tissues and Huh7 cells treated with hypoxia (1% O2) for 48 hours. We found that HCC patients with higher USP2-AS1 expression had a worse prognosis. We confirmed that lncRNA USP2-AS1 promoted the proliferation and invasion of HCC by enhancing YBX1-mediated HIF1α translation under hypoxia. In combination with USP2-AS1 knockdown, the efficacy of lenvatinib increased in a mouse model of subcutaneous liver cancer.

## Materials nd Methods

### Cells Culture

The human hepatoma cell lines MHCC97H, Huh7, PLC, MHCCLM3, Hep3B and human normal liver cells LO2 were from Liver Cancer Institute, Zhongshan Hospital, Fudan University. Cells were cultured in Dulbecco’s modified Eagle’s medium (DMEM High Glucose Pyruvate, Gibco) with 10% fetal bovine serum (FBS, Sigma) and 1% antibiotics in an incubator with 5% CO2 at 37°C. For hypoxic treatment, cells were cultured in a hypoxia incubator with 1% O2, 5% CO2, and 94% N2 for 48h, then were harvested. All cells used in this study have tested negative for mycoplasma.

### *In Vivo* Proliferation Assay

Five-week-old female BALB/c nude mice housed in a suitable environment according to the protocols approved by the Animal Ethics Committee of Zhongshan Hospital. 3×10^6^ Huh7 cells with USP2-AS1 overexpression or normal control after treated with hypoxia (1% O_2_) for 48h were subcutaneously injected into either side of the flank of each female nude mouse (n=4 mice per group). All mice were sacrificed after 4 weeks for tumor weight and volume measurement.

To explore the anti-tumor effect of lenvatinib combined with USP2-AS1 knockdown, the mice were randomly divided into two groups (n=4 mice per group), 3×10^6^ Huh7 cells with USP2-AS1 knockdown or Normal Contrast (NC) after treated with hypoxia (1% O_2_) for 48h were subcutaneously injected into either side of the flank of each female nude mouse. When the tumor volume exceeded 50 mm^3^, lenvatinib solvent diluted with PBS (22.6mg/kg per time for each nude mouse according to the manufacturer’s instructions) or PBS (as Normal Contrast) was administered by oral gavage, five times a week. Two weeks after the injection of lenvatinib, the mice were sacrificed for tumor weight and volume measurement.

### Protein Extraction and Immunoblotting

The cells were plated in a six-well plate and washed twice with PBS, then SDS-PAGE Sample Loading Buffer,1X 200uL (purchased from Shanghai Beyotime Biotechnology Company) was directly added to lyse the cells for protein extraction. The samples were then heated at 95°C for 10 minutes and then loaded onto a sodium dodecyl sulfate-polyacrylamide gel electrophoresis (SDS-PAGE) to separate proteins based on their different molecular weights. The gel was then transferred to a nitrocellulose membrane. The membrane was incubated with the corresponding primary and secondary antibodies, and the signals were visualized by ECL (Enhanced Chemiluminescence). The film was scanned by the ImageStudio system, photographed, and preserved for follow-up analysis.

### RNA Extraction and qRT-PCR

The RNA was extracted from cells or tissues with RNAeasy™ Animal RNA Isolation Kit with Spin Column (purchased from Shanghai Beyotime Biotechnology Company, China) according to the manufacturer’s protocol. Following the manufacturer’s instructions, Hifair^®^II 1st Strand cDNA Synthesis SuperMix (YEASEN Biotechnology, Shanghai, China) PCR premix was used for qRT-PCR. The gene expression level relative to GAPDH was calculated by the 2^-ΔΔCT^ method. Each experiment was performed in triplicates. The sequences of primers used in qRT-PCR are listed in **Supplementary Table 2**.

### Lentivirus, siRNA, and Cell Transfection

USP2-AS1 overexpression and knockdown lentiviral expression vectors are provided by Tsingke Biotechnology Co., Ltd (Beijing, China). Transfection was performed with corresponding transfection reagents when the cell density reached 30%-50%. 6-8h after transfection, the medium was changed, and puromycin was used for 48h for selection. After 2 weeks, a stably transfected cell line was obtained. YBX1-siRNAs and negative control siRNAs were provided by Ribobio Biotechnology Co., Ltd (Guangzhou, China). Viral transfections were carried out using the corresponding viral transfection reagents, Polyplus jetPRIME transfection reagents were used for all siRNAs transfection.

### Cell Proliferation and Colony Formation Assay

Cell proliferation assays were carried out using the BeyoClick™ EdU Cell Proliferation Kit with Alexa Fluor 555(EdU-555, Beyotime, China), according to the manufacturer’s protocols. In brief, 5000 cells were cultured in a 96-well plate. After the adherence of the cells, the 2 × Edu culture medium was added and incubated for 2 hours. After fixing, washing, and permeating, the configured Click Additive Solution was added and incubated at room temperature in the dark for 30 minutes. The nuclei were stained with Hoechst 33342 and incubated at room temperature in the dark for 10 minutes. Finally, the cells were observed and photographed using a fluorescence microscope.

### Clone Formation Assay

A total of 2000 cells were inoculated in a six-well plate. After the cells adhered to the wall, they were cultured in the anoxic incubator of 1% O_2_, and the culture medium was changed twice a week. Cells were harvested after 15 days. Each experiment was performed with three replicates.

### Migration and Invasion Assay

Cell migration and invasion assays were carried out using the Boyden chamber (BD Bioscience) with an aperture of 8um with or without Matrigel, and the experiments were performed according to the manufacturer’s protocols. The cells were washed with PBS and digested into a single-cell suspension. 1 × 10^5^ cells (suspended in 200ul medium containing 10%FBS) were added to the upper chamber and 600ul containing 30% FBS was added to the lower chamber and cultured in the hypoxia incubator for an appropriate time. The cells were fixed with 4% paraformaldehyde, stained with crystal violet, and washed by PBS. The cells on the surface of the upper chamber were observed and photographed using a microscope. The experiments were repeated three times.

### RNA-Protein Immunoprecipitation

RIP was performed by the MagnaRIPTM RNA-Binding Protein Immunoprecipitation Kit (Merck Millipore) according to the manufacturer’s protocols. 2 × 10^7^ cells were lysed by Complete RIP Lysis Buffer, centrifuged and the supernatant was incubated with magnetic beads conjugated to antibody (Five micrograms per analysis) against Y-box binding protein 1 (YBX1) at 4°C overnight. After the protein was digested by protease K, RNA was purified by phenol-chloroform. Finally, the qRT-PCR test was performed with specific primers for quantitative analysis of lncRNA USP2-AS1 and HIF1α mRNA.

### RNA Pulldown

LncRNA pulldown assay was performed by the Pierce™ Magnetic RNA-Protein PullDown Kit from Thermo according to the manufacturer’s protocols. Approximately 50 pmol of the RNA 3’ end desthiobiotinylation labeled lncRNA USP2-AS1 full-length or its truncated probes (synthesized by BersinBio, Guangzhou, China) were incubated with 50ul of streptavidin magnetic beads for 30 minutes at room temperature. The beads were then washed by using an equal volume of 20 mM Tris (pH 7.5). 100ul 1× RNA-protein binding buffer (Containing 100ug total protein) was added to the tube containing streptavidin magnetic beads and gently mixed and incubated at room temperature for 60 minutes. After washing the beads 1-2 times with an equal volume of 1x wash buffer (100ul), the beads were mixed with 50ul Elution Buffer and incubated at 37°C for 30 minutes. Supernatants were collected for silver staining or WB analysis.

### Tissue Microarrays and Liver Cancer Samples

HCC tissue microarrays were purchased from Shanghai Outdo Biotech Company (T16-339 TMA1, T16-339 TMA2, T16-T16-TMA3) for the FISH experiment. The clinical information was statistically analyzed based on the high and low USP2-AS1 positive cell rates. A total of 208 pairs of non-duplicate liver cancer paired samples and 216 liver cancer tissue samples (8 samples were repeated) were collected on the three tissue microarrays, of which the third tissue microarray had a small number of duplicate samples. These samples were included in the analysis of USP2-AS1 expression in normal liver and HCC tissues. In the survival and prognostic analysis, we excluded the duplicate samples. Two liver cancer samples used in the USP2-AS1 FISH experiment and 36 paired samples of HCC used in the qRT-PCR experiment came from Zhongshan Hospital Fudan University (approved by the Institutional Review Board (IRB) at Zhongshan Hospital Fudan University).

### RNA *In Situ* Hybridizations

LncRNA USP2-AS1 *in situ* hybridization kit was provided by Wuhan Servicebio technology Co., Ltd. Briefly, the dehydrated and fixed tissue microarray was dripped with a probe specific for lncRNA USP2-AS1 and hybridized overnight at 55°C in a thermostat. After hybridization, tissue microarray was washed by 2 × SSC at 37°C for 10 minutes, 1 × SSC at 37°C for 2 × 5 minutes and 0.5 × SSC for 10 minutes at room temperature, or formamide was added to wash nonspecific hybrids. Then the microarray was blocked with BSA and incubated with anti-DIG-488 at 37°C for 50 minutes, washed 4 times (5 minutes per wash). Finally, DAPI dye was added and incubated for 8 minutes, protected from light. After being washed, anti-fluorescence quenching tablets were added to seal. The rate of USP2-AS1 positive cells was counted using a fluorescence microscope.

### Related Web Tools

The protein-coding ability of USP2-AS1 has displayed by PhyloCSF (PhyloCSF in the UCSC Genome Browser (https://genome.ucsc.edu/). We use the lncATLAS (https://lncatlas.crg.eu/) to predict the location of USP2-AS1 in HCC cells. The binding site of USP2-AS1 with YBX1 was predicted through the online website catRAPID (http://service.tartaglialab.com/update_submission/380192/4af202c73a). We also used the RNAfold Webserver (http://rna.tbi.univie.ac.at/cgi-bin/RNAWebSuite/RNAfold.cgi) to predict the secondary structure of USP2-AS1. TCGA-LIHC (The Cancer Genome Atlas-Liver hepatocellular carcinoma) was used for verifying the overexpression of lncRNA USP2-AS1 in liver cancer and the correlation between YBX1 and HIF1A, YBX1 and USP2-AS1.

### Statistical Analysis

All experiments in this study were performed at least three times. Data were expressed as mean± SD. Differential comparisons of data between two groups were performed using paired or unpaired two-tailed t-tests. Statistical analysis was performed using GraphPad Prism 8.0.2 and R 4.0.5. The results were considered statistically significant when p < 0.05.

## Results

### USP2-AS1 Is Significantly Up-Regulated in Hepatocellular Carcinoma

**Figure 1:**
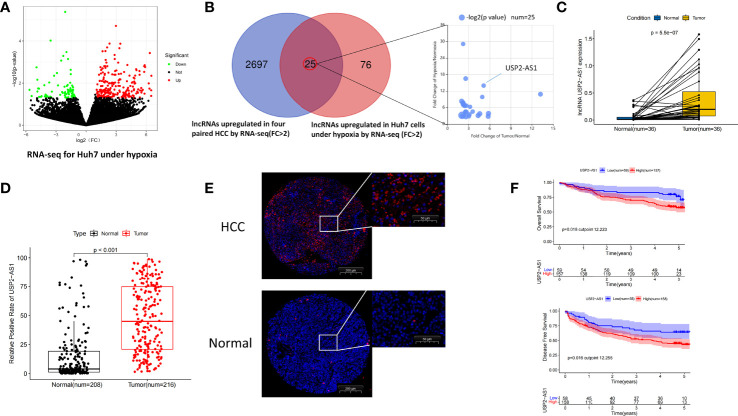
USP2-AS1 is significantly up-regulated in hepatocellular carcinoma. **(A)** Molecules differentially expressed under normoxia and hypoxia in Huh7 cells identified by RNA-seq. **(B)** 25 intersection candidate genes obtained by hypoxia-treated Huh7 cells and HCC tissue sequencing. **(C)** The relative expression of USP2-AS1 in 36 pairs of HCC and adjacent non-tumor specimens by RT-qPCR. **(D)** Relative expression of USP2-AS1 in the HCC chips by FISH experiments, unpaired Student’s t-test. **(E)** The relative positive cell rate of USP2-AS1 in the HCC chips by FISH experiments. **(F)** Kaplan–Meier analysis of overall survival time and disease-free survival time in HCC patients with high USP2-AS1 expression versus low USP2-AS1 expression.

To find hypoxia-associated lncRNAs with potential promoting effect on HCC, RNA sequencing analysis was performed using HCC tissues and adjacent normal tissues from 4 patients and HCC cell line Huh7 treated with hypoxia for 48h (**Figures 1A, B**). A total of 2722 lncRNAs were significantly up-regulated in HCC tissues, and 101 lncRNAs were up-regulated in hypoxia-treated Huh7 cells, the intersection between the two categories included 25 lncRNAs (**Figure 1B**).

Given that lncRNA USP2-AS1 (ubiquitin specific peptidase 2 antisense RNA 1) has the most pronounced fold change in the two categories, so we focused on the role of USP2-AS1 in HCC in our following studies. USP2-AS1 is a long non-coding RNA with 3798 nucleotides in length (USP2-AS1-208, ENST00000659432.1). Similar to other lncRNAs, we found USP2-AS1 has almost no protein-coding ability by PhyloCSF (**Figure S1A**). We also found that USP2-AS1 is likely entirely located in HCC cytoplasm by lncATLAS. (**Figure S1B**), which is confirmed by Fluorescent *in situ* hybridization (FISH) assay (**Figure S1C**).

To explore the relationship between USP2-AS1 and liver cancer, we analyzed the expression of USP2-AS1 in 36 pairs of HCC and adjacent non-tumor specimens by RT-qPCR. As expected, the expression of USP2-AS1 in HCC was significantly higher than in para-cancer tissues (**Figure 1C**). It was also confirmed in the FISH experiment using three tissue chips of human liver cancer (**Figures 1D, E**), which is consistent with the TCGA-LIHC (The Cancer Genome Atlas, Liver Hepatocellular Carcinoma) data (**Figure S1D**).

USP2-AS1 was differentially upregulated in hepatoma cell lines (Huh7, MHCC97H, HCCLM3, PLC) compared with normal human hepatocyte LO2 under both normoxic and hypoxic conditions (**Figures S2A, B**). We also found that the expression of USP2-AS1 was significantly up-regulated in these cell lines following 48h hypoxia (**Figure S2C**), which was consistent with our RNA sequencing results (**Figure 1A**). By analyzing the positive cell rate of USP2-AS1 in human liver cancer tissue microarrays and determining the cutoff value of USP2-AS1 by X-Tile software, we observed that patients with low expression of USP2-AS1 had better prognosis while patients with high expression of USP2-AS1 had shorter OS (Overall Survival) and DFS (Disease-Free Survival) time (**Figure 1F**).

Notably, clinical information of the tissue microarrays showed that USP2-AS1 remarkably correlated with tumor size and the TNM stage of HCC (**Table 1**). Importantly, ROC curve analysis showed lncRNA USP2-AS1 had a high AUC (Area Under Curve, 0.824) for HCC diagnosis (**Figure S2D**). The above results indicate that hypoxia-related lncRNA USP2-AS1 is up-regulated in HCC. USP2-AS1 positively correlated with worse outcomes in HCC patients and may be a potential prognostic tool.

**Table 1:** The clinic pathological factors of HCC patients.

Features	All cases	lncRNA USP2-AS1 exxpression		P-value
		Low	High	
Total number	216	59	157	
Age				0.7978
≤60	150	43	107	
>60	66	16	50	
Gender				0.6328
Male	170	49	121	
Female	46	10	36	
Liver cirrhosis				0.8716
with	89	26	63	
without	127	33	94	
Tumor size (cm)				0.0109*
≤5	89	34	55	
>5	127	25	102	
Tumor number				0.2343
solitary	198	51	147	
multiple	18	8	10	
Edmondson grade				0.8823
I + II	137	39	98	
III	79	20	59	
TNM stage				0.0098*
I + II	110	40	70	
III + IV	106	19	87	
HBsAg				0.6818
positive	102	25	77	
negative	114	34	20	
Recurrence				0.7515
with	144	37	107	
without	72	22	50	

*Statistical significant results.

### USP2-AS1 Enhances HCC Proliferation, Migration, and Invasion *In Vitro* Under Hypoxia

To investigate the role of USP2-AS1 in HCC, we constructed stable transfectants of MHCC97H and Huh7 cell lines overexpressing USP2-AS1 and knocked down USP2-AS1 in Huh7 cell lines by lentiviral transfection (**Figure 6A**). Under normoxic conditions, overexpression or knockdown of USP2-AS1 did not affect the proliferation, migration, and invasion ability of HCC (data not given). However, our previous RNA sequencing and FISH experiments have confirmed a significant difference in the expression of USP2-AS1 between liver cancer and para-cancer tissues (**Figures 1C–E**). And given that hypoxia treatment can increase USP2-AS1 expression in HCC cell lines (**Figure S2C**), we hypothesized USP2-AS1 promotes the progression of tumors by affecting tumor hypoxic metabolism.

**Figure 2:**
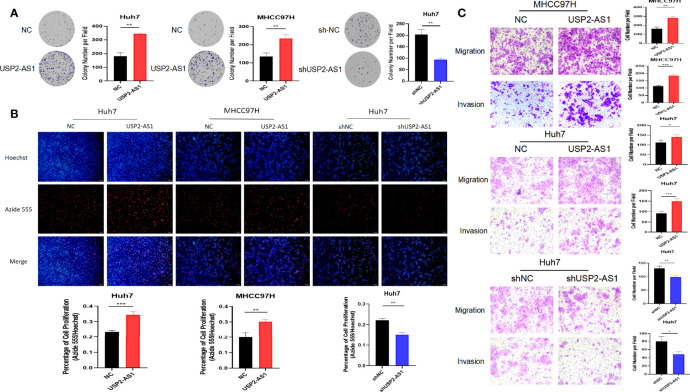
USP2-AS1 promotes HCC proliferation, migration, and invasion under hypoxia *in vitro*. **(A)** The effect of USP2-AS1 on the clonogenic capacity of HCC cell lines Huh7 and MHCC97H *in vitro* was verified under hypoxia. **(B)** EdU cell proliferation assays confirmed USP2-AS1 affected the proliferative capacity of hepatoma cell lines Huh7 and MHCC97H, under hypoxia, *in vitro*. **(C)** Overexpression or knockdown of USP2-AS1 under hypoxic conditions affected the migration and invasion ability of HCC cell lines Huh7 and MHCC97H *in vitro*. Two-tailed Student’s t-test, *p < 0.05, **p < 0.01, and ***p < 0.001.

After being treated with hypoxia for 48h, overexpression of USP2-AS1 dramatically increased HCC proliferation and clone formation *in vitro* (**Figures 2A, B**). Conversely, USP2-AS1 knockdown significantly inhibited the proliferation and clone formation ability of HCC (**Figures 2A, B**). We observed that the migration and invasion of HCC cells decreased after knockdown of USP2-AS1, while the reverse was observed with USP2-AS1 overexpression (**Figure 2C**). These results indicate that USP2-AS1 significantly enhances the proliferation, migration, and invasion of liver tumor cells *in vitro* under hypoxia.

### USP2-AS1 Physically Interacts With YBX1 Under Hypoxia

**Figure 3:**
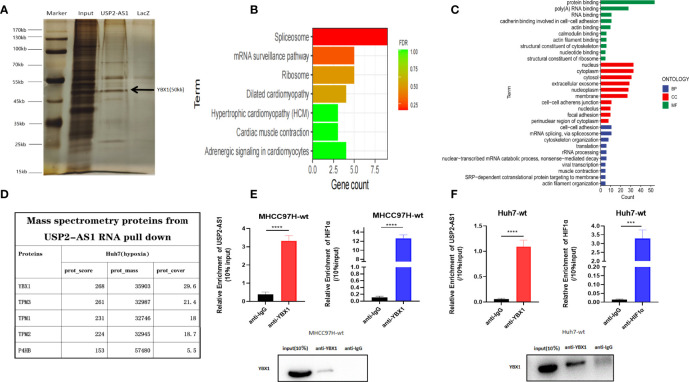
USP2-AS1 physically interacts with YBX1 under hypoxia. **(A)** Silver staining and mass spectrometry analysis following RNA pull-down of USP2-AS1 or Laz (as negative control probe) in Huh7 cells after exposed to hypoxia for 48 hours. **(B)** KEGG pathway enrichment analysis for differential protein molecules from mass spectrometry analysis. **(C)** GO enrichment analysis for differential protein molecules from mass spectrometry analysis. **(D)** The top 5 differential proteins from mass spectrometry analysis. **(E)** YBX1 associated HIF1α and USP2-AS1 as measured by qRT-PCR following RIP YBX1 in MHCC97H cells. **(F)** YBX1 associated HIF1α and USP2-AS1 as measured by qRT-PCR following RIP YBX1 in Huh7 cells after being treated with hypoxia for 48 hours. ***p < 0.001, ****p < 0.0001.

To understand the mechanism of USP2-AS1 in HCC progression under hypoxia, we performed RNA pulldown assays on Huh7 cells cultured in hypoxia for 48 hours (**Figures 3A** and **S3A, B**). A total of 75 proteins were identified as potential molecules that may bind to USP2-AS1 by using mass spectrometry (**Table S1**). We conducted GO enrichment analysis as well as KEGG pathway enrichment analysis of these 75 protein molecules. GO enrichment results showed that MF (Molecular Function) principally included protein binding and RNA binding, BP (Biological Process) mainly comprised of mRNA Splicing, cell-cell adhesion, and translation, and CC (Cellular Component) was primarily in the cytoplasm and nucleus (**Figures 3B, C**). Among these protein molecules, we noticed that YBX1(Y-box binding protein 1) is highly likely to bind to USP2-AS1 with the highest protein spectrum score (**Figure 3D**). The binding of YBX1 to USP2-AS1 was confirmed by YBX1-RIP assay in the MHCC97H cell line (**Figure 3E**), and in the Huh7 cell line (**Figure 3F**).

**Figure 4:**
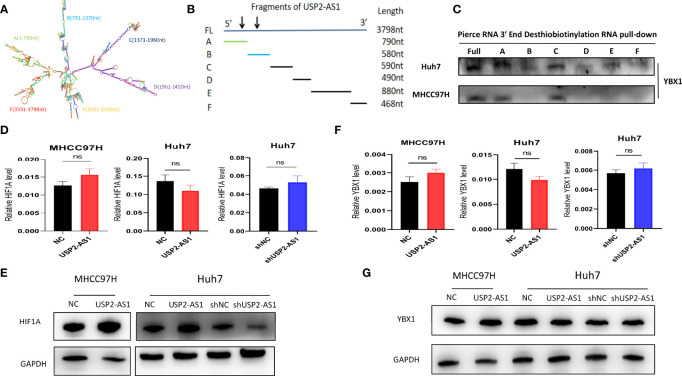
USP2-AS1 increases HIF1α translation under hypoxia. **(A)** The secondary structure of USP2-AS1 was predicted by RNAfold Webserver. **(B)** Six truncated probes (**A–F** fragments) of USP2-AS1 were designed to perform RNA pulldown assays. **(C)** USP2-AS1 physically interacts with YBX1 by the A and C fragments under hypoxia measured by WB. **(D)** USP2-AS1 overexpression or knowdown failed to cause significant changes in HIF1α mRNA measured by qRT-PCR. **(E)** USP2-AS1 affects HIF1α protein levels, as measured by WB. **(F)** USP2-AS1 overexpression or knowdown failed to cause significant changes in YBX1 mRNA measured by qRT-PCR. **(G)** USP2-AS1 overexpression or knockdown does not affect YBX1 protein levels compared with the controls, ns means not significant.

To investigate how USP2-AS1 binds to YBX1, we predicted the binding site of USP2-AS1 with YBX1 through the online website catRAPID It indicated the segment 500nt-1000nt of USP2-AS1 was likely to bind to YBX1 (**Figure S2E**). We also found USP2-AS1 contains 6 fragments with specific secondary structures by the RNAfold Webserver (**Figure 4A**). We hypothesized that these secondary structures (A-F fragments) are the binding sites of USP2-AS1 with YBX1. We designed six truncated probes (corresponding to A-F fragments, respectively) of USP2-AS1, such as A (1-790nt), B (791-1370nt), C (1371-1960nt), D (1961-2450nt), E (2451-3330nt) and F (3331-3798nt) for RNA pulldown assays (**Figures 4B** and **S3B, C**). We demonstrated that fragment A and C are necessary to mediate the binding of USP2-AS1 and YBX1 (**Figure 4C**). These results show USP2-AS1 physically interacts with YBX1 by the A and C fragments under hypoxia.

### USP2-AS1 Enhances HIF1α Translation Under Hypoxia

Hypoxia-inducible factors (HIFs) are critical transcriptional activators mediating tumor hypoxia metabolism, and their imbalance plays an important role in tumor development (27), so we explored whether the expression of USP2-AS1 affects the levels of HIF1α under hypoxia. However, the overexpression of USP2-AS1 did not affect the levels of HIF1α mRNA in Huh7 or MHCC97H cells, and the knockdown of USP2-AS1 also did not affect the HIF1α mRNA in Huh7 (**Figure 4D**). Interestingly, we found that overexpression of USP2-AS1 increased HIF1α protein levels under hypoxia while USP2-AS1 knockdown reduced the protein levels of HIF1α compared with the control group (**Figures 4E** and **S4A**). However, no changes in HIF2α mRNA and protein levels were observed after knockdown or overexpression of USP2-AS1 (data not shown), indicating that the effect of USP2-AS1 on HIF1α protein levels is specific.

Previous studies have indicated that YBX1 promotes the translation of HIF1α protein by directly binding to the 5’ UTR of HIF1α mRNA to mediate the metastasis of osteosarcoma (28). At the same time, it indicated that the expression of YBX1 and HIF1α showed a significant positive correlation in the TCGA-LIHC (**Figure S4B**), which is consistent with previous studies. In this study, we confirmed USP2-AS1 binds to YBX1, but whether USP2-AS1 changed the protein levels of HIF1α by affecting the transcription or translation level of YBX1 remains unclear. We found a positive correlation of expression between YBX1 and USP2-AS1 in TCGA-LIHC (**Figure S4C**), so we hypothesized USP2-AS1 may increase HIF1α protein levels by affecting the expression of YBX1. Unfortunately, We further demonstrated that overexpression or knockdown of USP2-AS1 failed to cause significant changes in YBX1 mRNA or protein levels (**Figures 4F, G** and **S4D**). These results suggested that USP2-AS1 did alter the protein levels of HIF1α under hypoxia, but not affected the transcriptional or translation levels of YBX1.

### USP2-AS1 Enhances YBX1 Binding to HIF1α mRNA Under Hypoxia

**Figure 5:**
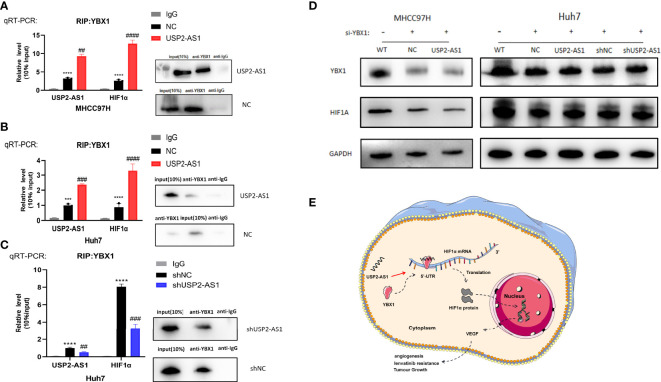
USP2-AS1 enhances YBX1 protein binding to HIF1α mRNA under hypoxic conditions. **(A)** RIP assays were performed to confirm that USP2-AS1 overexpression in MHCC97H can increase the effective binding of YBX1 to HIF1α mRNA. **(B)** RIP assays showed that USP2-AS1 overexpression in Huh7 increases the binding of YBX1 protein to HIF1α mRNA. **(C)** USP2-AS1 knockdown in Huh7 significantly inhibits the binding of YBX1 protein to HIF1α mRNA by RIP assays. HIF1α mRNA and USP2-AS1 were measured by RT-qPCR after RIP. WB assays were performed to test the experiment quality (10%input used as a positive control and IgG was the negative control). **(D)** WB assays were performed to analyze the effect of USP2-AS1 on HIF1α protein in MHCC97H and Huh7 after YBX1 was knocked down by siRNA, GAPDH as protein loading controls, WT as the controls. **(E)** The possible mechanism of USP2-AS1 regulating HIF1α protein translation by YBX1 under hypoxia in HCC, ^##^p < 0.01, and *** or ^###^p < 0.001, **** or ^####^p < 0.0001.

LncRNAs can inhibit or promote the translation of HIF1α by binding to YBX1 protein without changing the expression level of YBX1 protein itself (29–31). We investigated whether USP2-AS1 could enhance the effect of YBX1 on the translation of HIF1α protein by binding to YBX1. RIP experimental results indicated HIF1α mRNA did bind to YBX1 protein (**Figures 3E, F**), which was consistent with the previous outcomes. We observed that under hypoxic conditions, overexpression of USP2-AS1 increased the binding efficiency of YBX1 to USP2-AS1 and significantly increased the effective binding of YBX1 to HIF1α mRNA (**Figures 5A, B**). The knockdown of USP2-AS1 had the opposite effect on the binding level of YBX1 and HIF1α mRNA (**Figure 5C**).

We used small interference RNA to change the expression of YBX1 in USP2-AS1 overexpression or knockdown cell lines (**Figure 5D**). The effect of overexpression or knockdown of USP2-AS1 on the translation level of HIF1α protein under hypoxia was significantly abrogated after the knockdown of YBX1 (**Figure 5D**). Cell proliferation, migration, and invasion experiments also showed that knockdown of YBX1 eliminated the effect of USP2-AS1 on the growth of HCC cells (**Figures S5A, B**, **S6A, B**). The results suggest that USP2-AS1 can increase the protein levels of HIF1α by enhancing YBX1 protein binding to HIF1α mRNA under hypoxia, ultimately promoting the growth and development of HCC.

### USP2-AS1 Knockdown Enhances the Efficacy of Lenvatinib on HCC in Mice Tumor Xenograft Model

**Figure 6:**
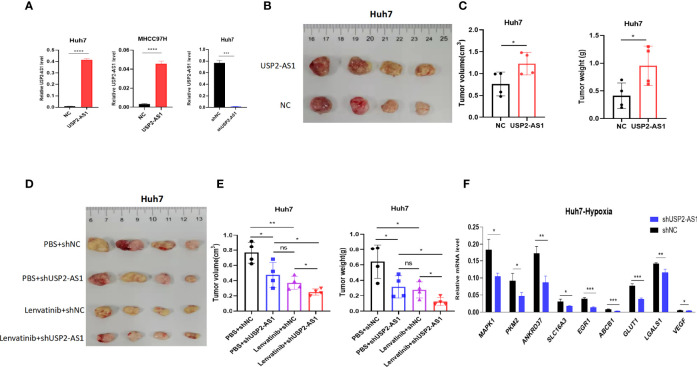
USP2-AS1 knockdown can increase the efficacy of lenvatinib in mice tumor xenograft model. **(A)** Construction of Huh7 and MHCC97H cell lines overexpressing USP2-AS1 and Huh7 USP2-AS1 knockdown cell lines. **(B, C)** Tumor volume and tumor weight of NC (Normal Contrast) or USP2-AS1 overexpressing Huh7 cells in the xenograft mouse model (n = 4). **(D, E)** Tumor volume and tumor weights of NC or USP2-AS1 overexpressing Huh7 cells in a xenograft mouse model (n = 4) with PBS or Lenvatinib. **(F)** Several downstream target genes of HIF1α were down-regulated following USP2-AS1 knockdown in Huh7 cell under hypoxia. Two-tailed Student’s t-test, *p < 0.05, **p < 0.01, ***p < 0.001, ****p < 0.0001, and ns, no statistically significant differences.

To further explore the role of USP2-AS1 acting on the growth of HCC *in vivo*, we constructed tumor xenograft model in mice by using USP2-AS1 overexpression or knockdown Huh7 cell line. The tumor xenograft model showed that overexpression of USP2-AS1 promoted the growth of tumor while USP2-AS1 knockdown inhibited the proliferation of HCC *in vivo* (**Figures 6B–E**). These results suggest USP2-AS1 overexpression or knockdown can affect the growth of tumor in our mice tumor xenograft model.

Hypoxia often mediates the resistance of solid tumors to chemotherapeutic drugs. Lenvatinib is a multikinase inhibitor like sorafenib, which was developed in Japan as a multitargeted kinase inhibitor for the treatment of advanced-stage HCC in 2018. Lenvatinib significantly improved the overall survival and progression-free survival time of patients with advanced HCC compared with sorafenib treatment (32). Sorafenib treatment is also limited by drug resistance. Under hypoxic conditions, transcription factor HIF1α can mediate the transcriptional activation of some genes related to glucose uptake, metabolism, and cell proliferation, such as MAPK1, VEGF, MDR1 (ABCB1), GLUT1, to facilitate the adaptive response of liver tumor cells to hypoxia, thereby increasing the therapeutic resistance of HCC to sorafinib (14). Therefore, the therapeutic effect of sorafenib is increased in liver cancer when it is combined with HIF1α inhibitors (33, 34). However, the mechanisms of HCC resistance to lenvatinib are poorly understood.

Our previous study showed that USP2-AS1 increased the protein levels of HIF1α in an anoxic environment, RT-qPCR also showed that the expression of target genes downstream of HIF1α was also significantly down-regulated in USP2-AS1 knockdown Huh7 cell line (**Figure 6F**), suggesting that USP2-AS1 knockdown may enhance the efficacy of lenvatinib. Therefore, we further explored whether USP2-AS1 knockdown enhanced the therapeutic effect of lenvatinib on HCC in mice tumor xenograft model. We observed that the tumor volume and tumor weights of mice treated with lenvatinib were significantly lower than those of the control group in the HCC xenograft model (**Figures 6D, E**). Importantly, compared with control groups, the tumors’ weight and volume were the smallest in the group treated with lenvatinib and USP2-AS1 knockdown (**Figures 6D, E**), indicating that USP2-AS1 knockdown can enhance the therapeutic effect of lenvatinib in HCC in our mice tumor xenograft model.

## Discussion

Although a large number of studies mainly focus on protein-coding genes and their anti-tumor roles, recent studies have shown that mutations and abnormal expression of non-coding genes, especially long non-coding RNA play an important role in tumor development (35). Exploring the specific molecular mechanisms of lncRNAs in cancer is instrumental in understanding the complex process of cancer occurrence and development, and provides promising targets for tumor drug treatment.

Our study identified a hypoxia-associated lncRNA USP2-AS1.And we observed USP2-AS1 was significantly up-regulated in HCC compared with normal liver tissue, patients with low expression of USP2-AS1 had better prognosis while patients with high expression of USP2-AS1 had shorter OS and DFS time. Moreover, clinical information showed that USP2-AS1 related with tumor size and the TNM stage of HCC. And ROC curve analysis showed USP2-AS1 had a potential for HCC diagnosis. *In vitro* and *in vivo* results, USP2-AS1 knockdown significantly inhibited the growth of HCC, while USP2-AS1 overexpression promoted the proliferation, migration, and invasion ability of liver cancer cells.

In addition, we confirmed that USP2-AS1 increases the level of HIF1α protein, by enhancing HIF1α mRNA binding to YBX1 protein under hypoxia. We also observed that USP2-AS1 knockdown decreased the transcript level of some downstream target genes of HIF1α under hypoxia, such as MAPK1, VEGF, MDR1(ABCB1), GLUT1, whose transcriptional activation play an important role in tumor hypoxia metabolism (36–39). These results suggest that USP2-AS1 promotes the growth and development of HCC by increasing the expression of HIF1α proteins and its downstream target genes.

The mechanism of tumor development is complex and different tumors have unique metabolic processes and tumor micro-environments. Solid tumors often suffer from hypoxia due to rapidly growing cells. Hypoxia-related factors or metabolic pathways are activated by tumor cells to adapt to the hypoxic environment (40). HIF1α, which is significantly up-regulated under hypoxia, is a critical regulatory factor of hypoxia metabolism in tumor cells. It contributes to the development of tumors under hypoxia by promoting the transcription of many downstream target genes related to hypoxia (41). Hydroxylation, acetylation, ubiquitination, and phosphorylation regulate the transcriptional activity of HIF1α (42), but there are few direct regulatory mechanisms of its translation level. However, it has been shown that certain RNA binding proteins, such as YBX1, can directly bind to HIF1α mRNA to regulate its protein translation (28). Our study observed hypoxia-associated lncRNA USP2-AS1 binds to YBX1 protein to enhance its binding to HIF1α mRNA and directly promotes the protein translation of HIF1α (**Figure 5E**).

In recent years, some lncRNAs mediating the translation of HIF1α through YBX1 have been reported. For example, the PERK/eIF2 signal pathway inhibits the expression of downstream target genes of HIF1α *via* YBX1-dependent regulation of HIF1α translation (29). LncRNA SNHG6 promotes carcinogenesis by enhancing the translation of YBX1-mediated HIF1α in clear cell renal cell carcinoma (31). LncRNA HITT can reduce HIF1α protein translation through YBX1 in colorectal tumors and cervical cancer while forming a feedback regulatory loop with HIF1α to regulate angiogenesis and tumor growth (30). However, the direct effect of hypoxia-associated lncRNAs on the protein translation of HIF1α in HCC has not been reported. Despite USP2-AS1 is up-regulated under hypoxia, USP2-AS1 specifically increases the protein level of HIF1α but not HIF2α, suggesting that the molecular mechanisms of hypoxia metabolism in tumor growth are complex and need further research.

The resistance of HCC to drug treatment is an important reason for the poor prognosis of HCC patients. The mechanisms of drug resistance in HCC therapy are complex (43, 44). Transcriptional activation of molecules such as VEGF induced by HIF1α under hypoxia is one of the most important mechanisms that mediate drug resistance to lenvatinib in HCC. Alternatively, it has been reported that EGFR-activated mutant NSCLC cells change the regulation of VEGF. In EGFR mutated cells, EGFR is the main regulator of HIF1α and VEGF (45). Patients with hypoxic tumors may benefit from EGFR inhibitors already available in the clinic (46). Also, it has been proved that PLAGL2-EGFR-HIF1/2A signaling pathway promotes the progression of HCC and affects the response of cancer cells to anti-EGFR drug erlotinib (47). These studies suggest complex relationships exist between HIF1α, VEGF, and EGFR, so the combination of one or more inhibitors of HIF1α, VEGR, EGFR with lenvatinib may enhance their therapeutic effect in hepatocellular carcinoma (48). The hypoxia-associated lncRNA USP2-AS1 knockdown significantly inhibited the translation of HIF1α protein and the transcriptional activation of its downstream target gene, such as VEGF, and increased the inhibitory effect of lenvatinib on the growth of HCC mice xenograft tumors. So, the combination of HIF1α inhibitors and lenvatinib may hold therapeutic potential in HCC.

In summary, our study identified a hypoxia-associated lncRNA USP2-AS1, which promotes the growth of hepatocellular carcinoma under hypoxia. In mechanism, USP2-AS1 physically interacts with YBX1 to increase the protein translation of HIF1α under hypoxia. We also found that the USP2-AS1 knockdown can enhance the inhibitory effect of lenvatinib on the growth of HCC in our mice tumor xenograft model.

## Data Availability Statement

The datasets presented in this study can be found in online repositories. The names of the repository/repositories and accession number(s) can be found in the article/**Supplementary Material**.

## Ethics Statement 

The studies involving human participants were reviewed and approved by Ethics Committee of Zhongshan Hospital affiliated to Fudan University. The patients/participants provided their written informed consent to participate in this study. The animal study was reviewed and approved by Animal Ethics Committee of Fudan University.

## Author Contributions

S-PC and G-QZ conceived and designed the study. L-NS and X-XX collected the data, analyzed the data, and wrote the manuscript. J-LW, J-XD and J-LC assisted with the data analyses and participated in the writing of manuscript. ZD and JZ coordinated the study. All authors read and approved the final manuscript. All authors contributed to the article and approved the submitted version.

## Funding

This study was jointly supported by the National Key R&D Program of China (2019YFC1315800, 2019YFC1315802), National Natural Science Foundation of China (No.82150004, 81830102, 81772578, 81802991), Fudan University and Shanghai Municipal Key Clinical Specialty (IDF152064/014), The Leading Project of the Science and Technology Committee of Shanghai Municipality (Grant number: No. 21Y21900100).

## Conflict of Interest

The authors declare that the research was conducted in the absence of any commercial or financial relationships that could be construed as a potential conflict of interest.

## Publisher’s Note

All claims expressed in this article are solely those of the authors and do not necessarily represent those of their affiliated organizations, or those of the publisher, the editors and the reviewers. Any product that may be evaluated in this article, or claim that may be made by its manufacturer, is not guaranteed or endorsed by the publisher.

## Abbreviations

HCC, Hepatocellular carcinoma; OS, Overall survival; TME, Tumor microenvironment; DFS, Disease-free survival; USP2-AS1, ubiquitin specific peptidase 2 antisense RNA 1; RIP, RNA immunoprecipitation; TCGA, The Cancer Genome Atlas; YBX1, Y-box binding protein 1; HIF1α, hypoxia inducible factor 1 subunit alpha; FISH, Fluorescent *in situ* hybridization; Edu,5-Ethynyl-2’-deoxyuridine; ECL, Enhanced Chemiluminescence; LIHC, Liver Hepatocellular Carcinoma; GO, Gene oncology; NC, Normal Contrast.
