# Supplementary material for: LncRNA USP2-AS1 Promotes Hepatocellular Carcinoma Growth by Enhancing YBX1-Mediated HIF1α Protein Translation Under Hypoxia
Source: Front Oncol. 2022 May 25;12:882372. doi: 10.3389/fonc.2022.882372 (PMC9174509; doi:10.3389/fonc.2022.882372)
Supplement: Supplementary file 1 [file DataSheet_1.pdf]

Supplementary information (Supplementary Figures and Tables)

LncRNA USP2-AS1 promotes hepatocellular carcinoma growth by enhancing YBX1-mediated HIF1α protein translation under hypoxia

Shi-Ping Chen, Gui-Qi Zhu, Xiao-Xia Xing, Jing-Lei Wan, Jia-Liang Cai, Jun-Xian Du, Li-Na Song, Zhi Dai, Jian Zhou

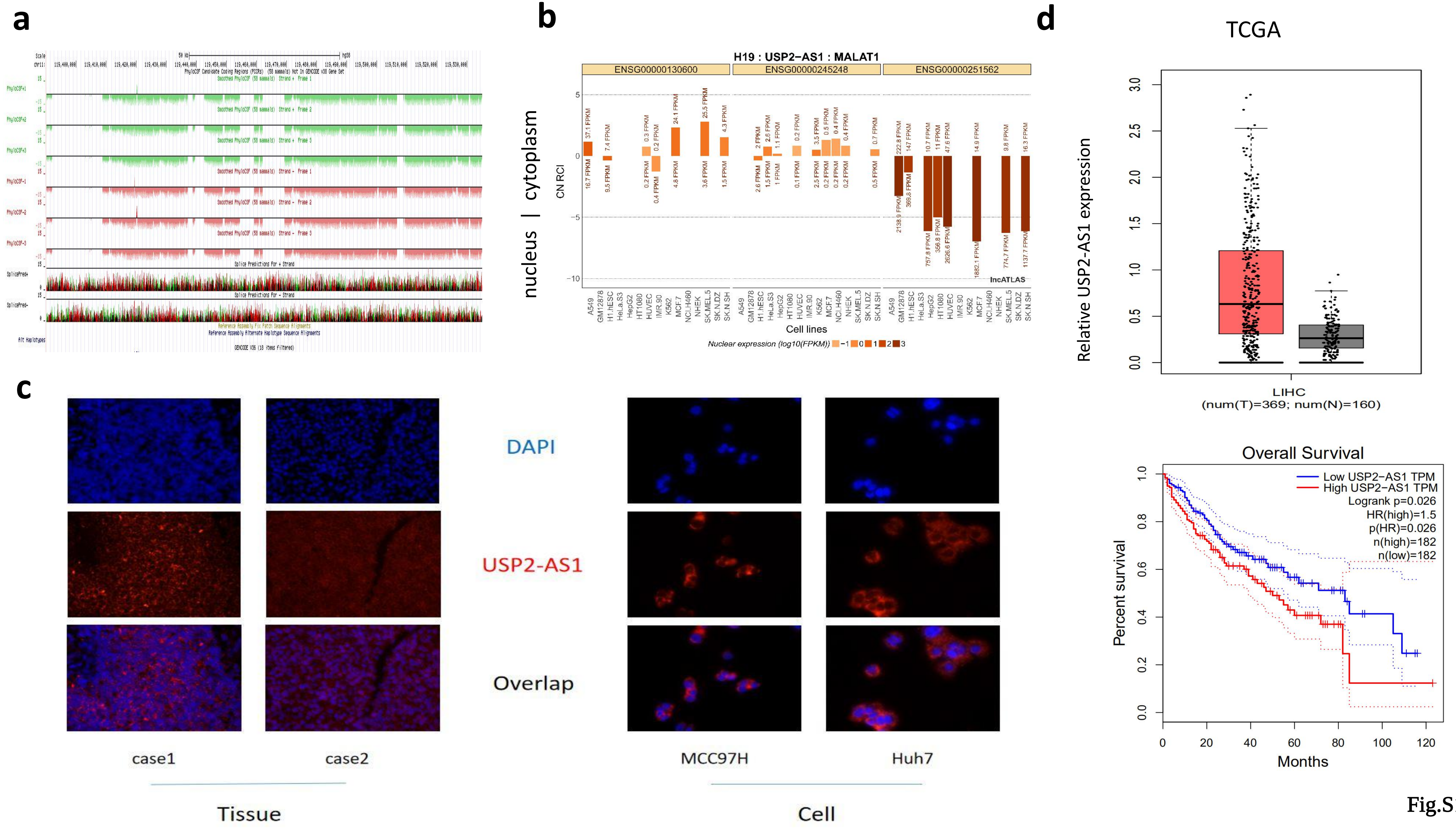
a
b
H19 : USP2−AS1 : MALAT1
ENSG00000130600
ENSG00000245248
ENSG00000251562
nucleus | cytoplasm
CN RCI
Cell lines
lncATLAS
Nuclear expression (log10(FPKM))
c
DAPI
USP2-AS1
Overlap
case1
case2
Tissue
MCC97H
Huh7
Cell
d
TCGA
Relative USP2-AS1 expression
LIHC
(num(T)=369; num(N)=160)
Overall Survival
Low USP2−AS1 TPM
High USP2−AS1 TPM
Logrank p=0.026
HR(high)=1.5
p(HR)=0.026
n(high)=182
n(low)=182
Percent survival
Months

Fig.S1.

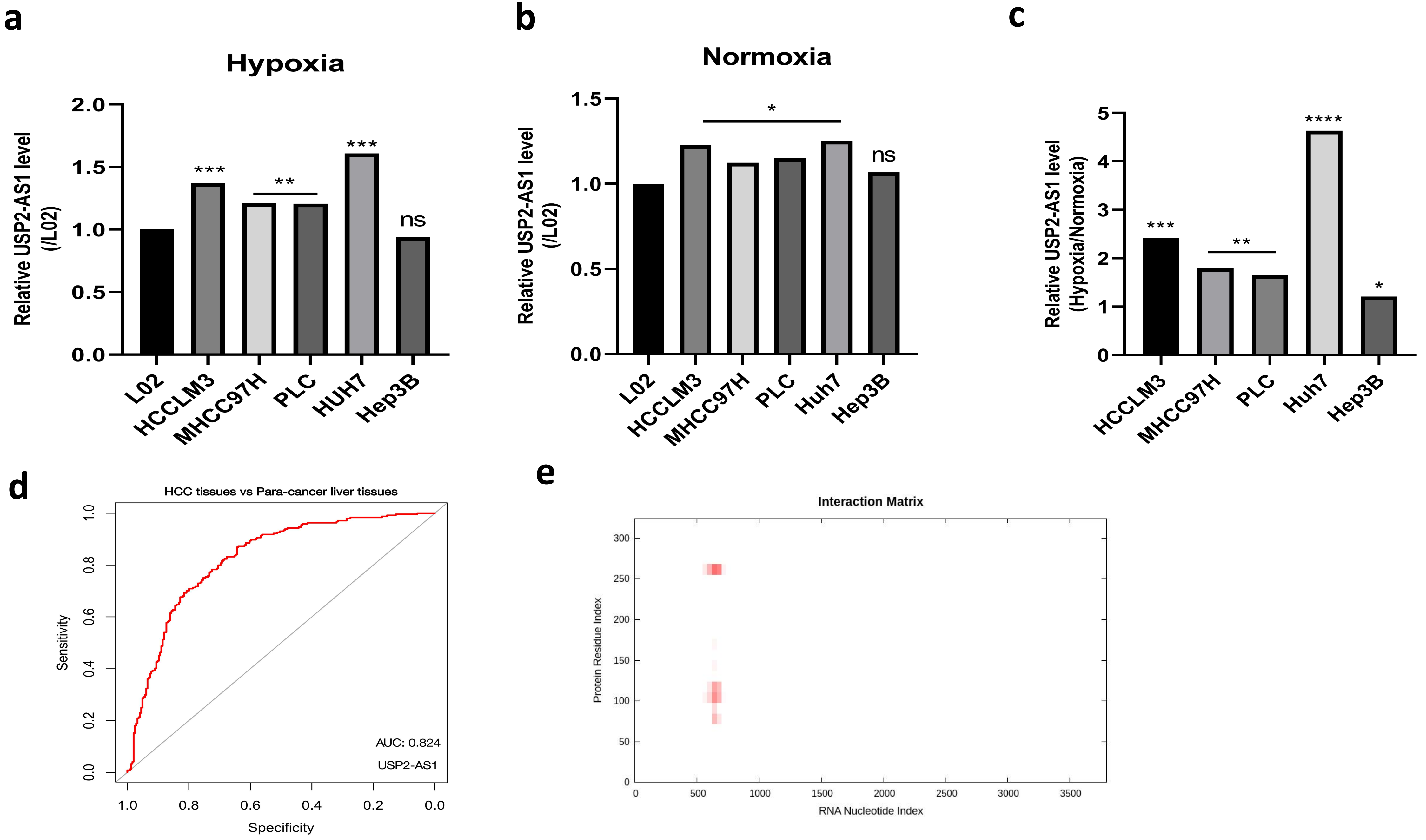

a
Hypoxia
Relative USP2-AS1 level (/L02)
2.0
1.5
1.0
0.5
0.0
***
**
***
ns
L02
HCCLM3
MHCC97H
PLC
HUH7
Hep3B
b
Normoxia
Relative USP2-AS1 level (/L02)
1.5
1.0
0.5
0.0
*
ns
L02
HCCLM3
MHCC97H
PLC
Huh7
Hep3B
c
Relative USP2-AS1 level (Hypoxia/Normoxia)
5
4
3
2
1
0
***
**
****
*
HCCLM3
MHCC97H
PLC
Huh7
Hep3B
d
HCC tissues vs Para-cancer liver tissues
Sensitivity
Specificity
1.0
0.8
0.6
0.4
0.2
0.0
AUC: 0.824
USP2-AS1
e
Interaction Matrix
Protein Residue Index
RNA Nucleotide Index
300
250
200
150
100
50
0
500
1000
1500
2000
2500
3000
3500

Fig.S2.

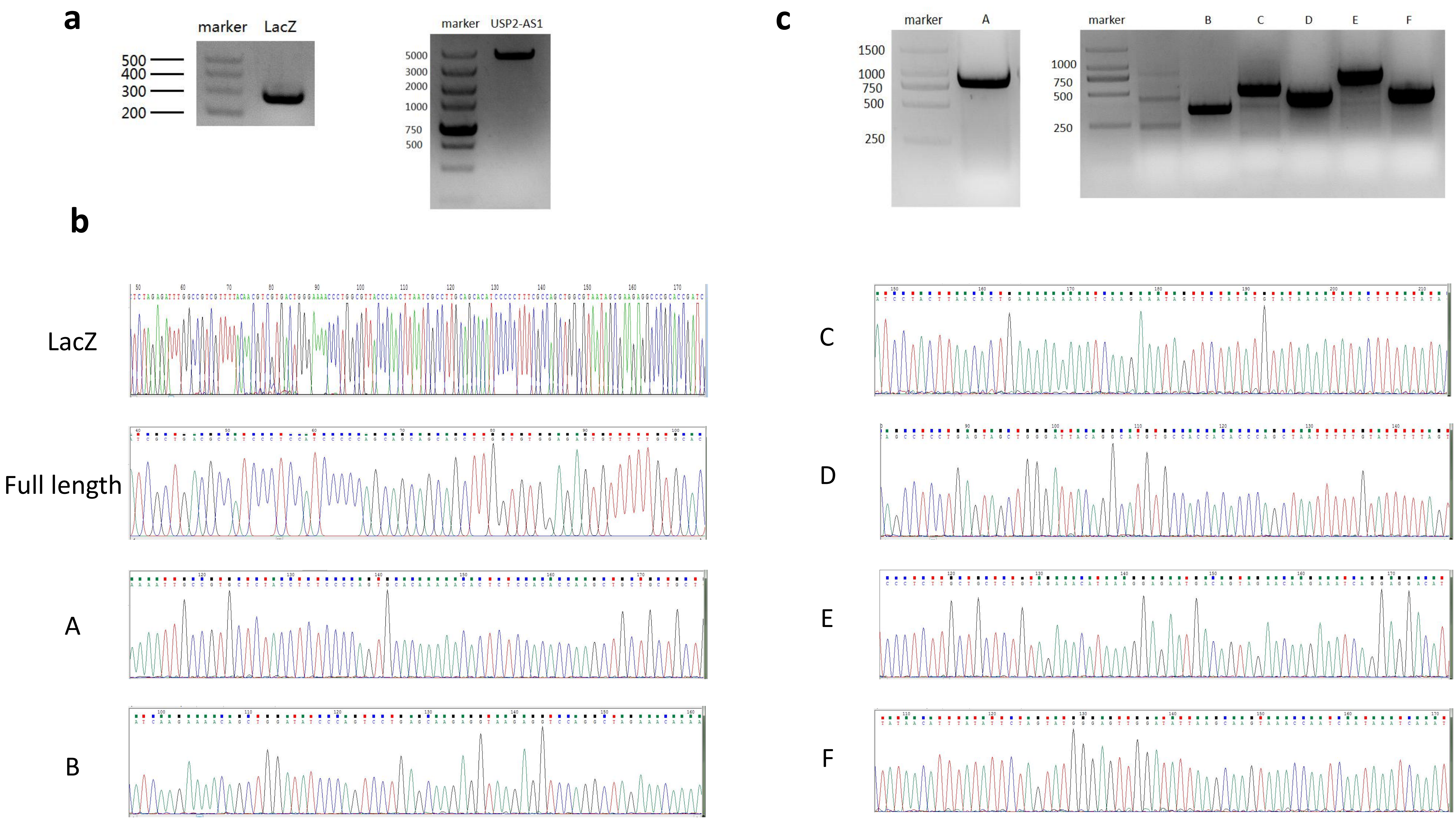
a
marker
LacZ
500
400
300
200
marker
USP2-AS1
5000
3000
2000
1000
750
500
c
marker
A
1500
1000
750
500
250
marker
B
C
D
E
F
1000
750
500
250
b
LacZ
Full length
A
B
C
D
E
F

Fig.S3.

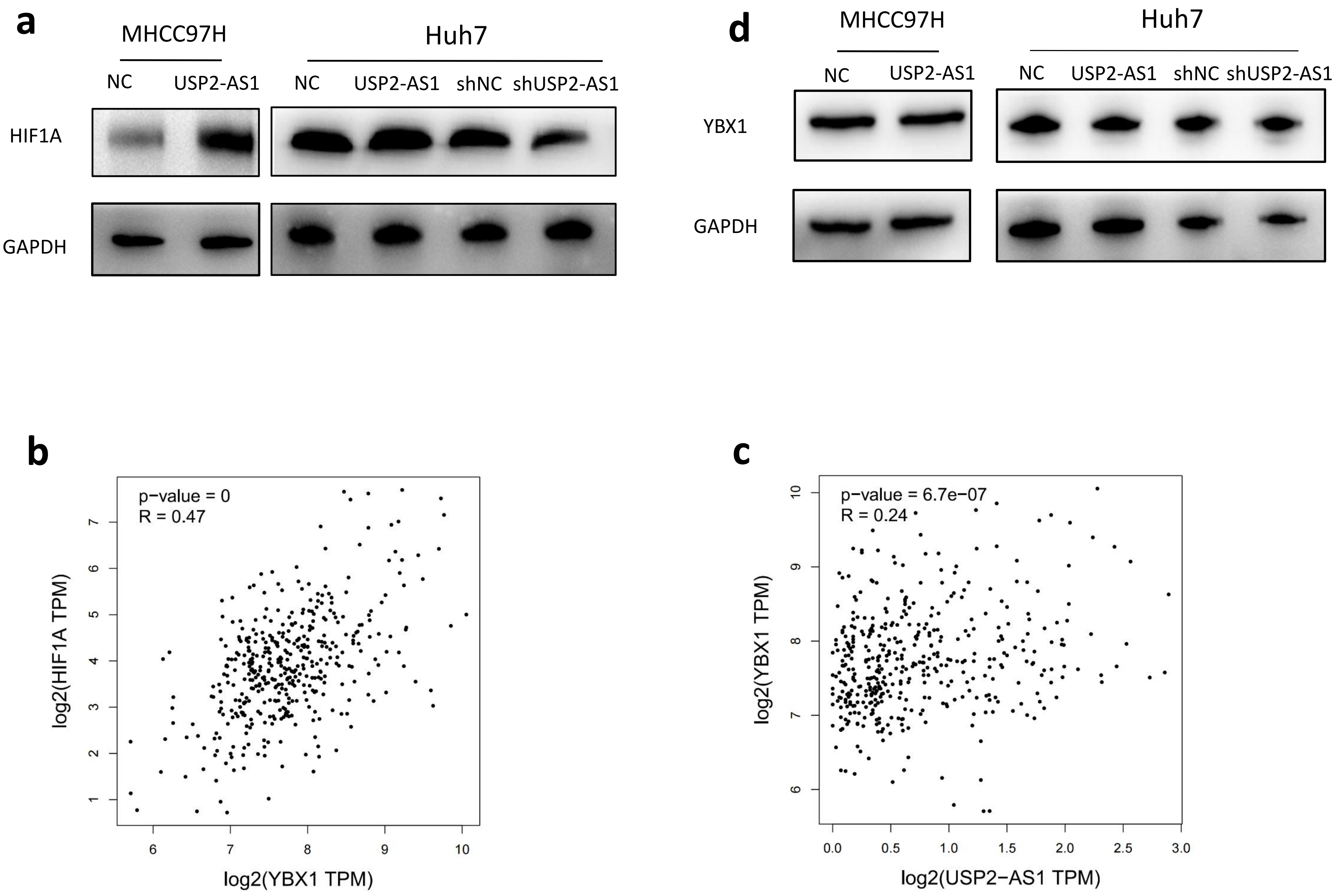
a
MHCC97H
Huh7
NC
USP2-AS1
NC
USP2-AS1
shNC
shUSP2-AS1
HIF1A
GAPDH
d
MHCC97H
Huh7
NC
USP2-AS1
NC
USP2-AS1
shNC
shUSP2-AS1
YBX1
GAPDH
b
p−value = 0
R = 0.47
log2(HIF1A TPM)
log2(YBX1 TPM)
c
p−value = 6.7e−07
R = 0.24
log2(YBX1 TPM)
log2(USP2−AS1 TPM)

Fig.S4.

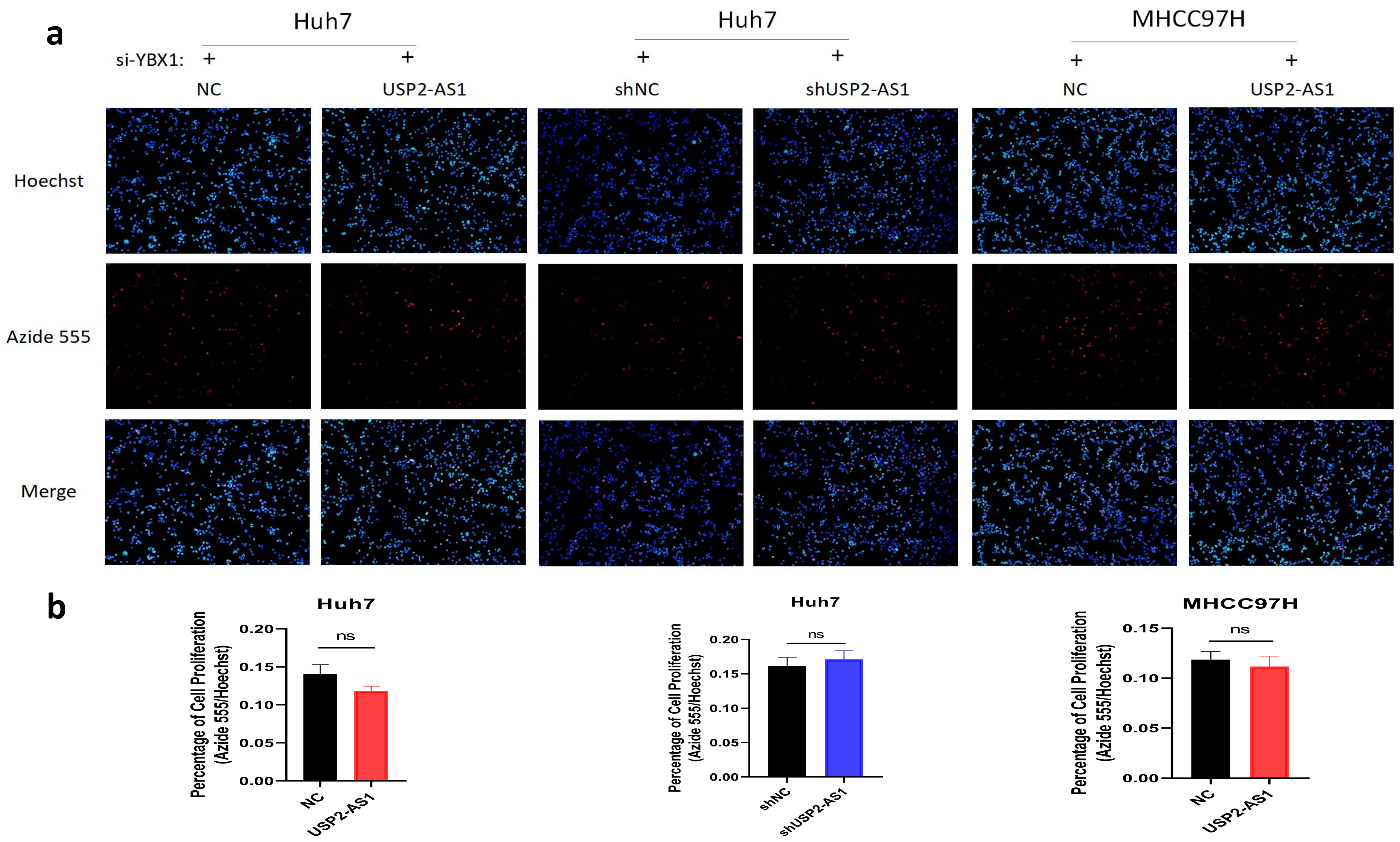
a
Huh7
Huh7
MHCC97H
si-YBX1:
+
NC
USP2-AS1
shNC
shUSP2-AS1
Hoechst
Azide 555
Merge
b
Huh7
MHCC97H
Percentage of Cell Proliferation (Azide 555/Hoechst)
0.20
0.15
0.10
0.05
0.00
ns
NC
USP2-AS1
shNC
shUSP2-AS1

Fig.S5.

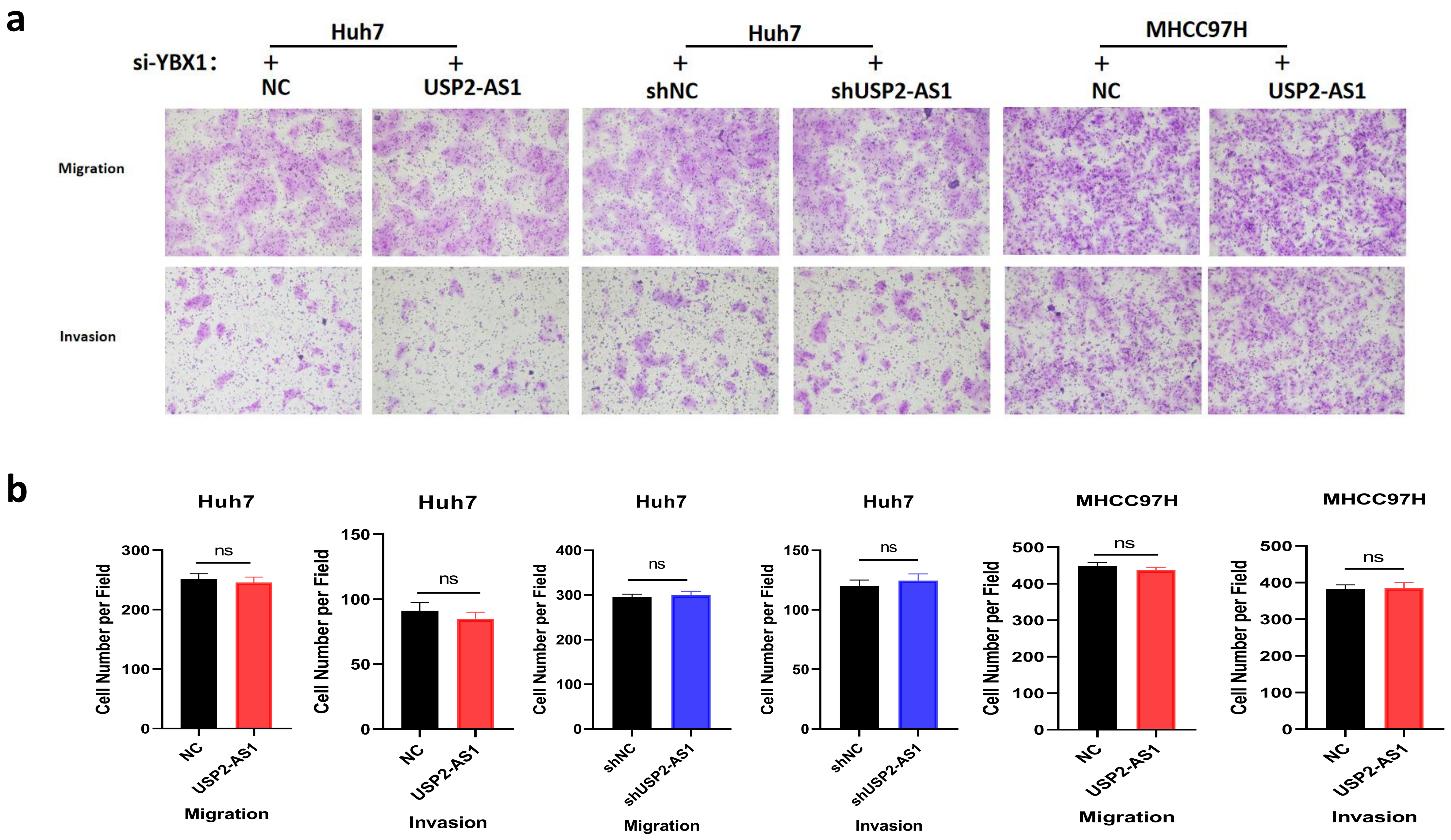
a
Huh7
Huh7
MHCC97H
si-YBX1：
+
NC
+
USP2-AS1
+
shNC
+
shUSP2-AS1
+
NC
+
USP2-AS1
Migration
Invasion
b
Huh7
Huh7
Huh7
Huh7
MHCC97H
MHCC97H
Cell Number per Field
ns
NC
USP2-AS1
shNC
shUSP2-AS1
Migration
Invasion

Fig.S6.

Supplementary Figure legends:

Fig.S1. The expression and localization of USP2-AS1. **a** USP2-AS1 has almost no protein-coding, viewed by PhyloCSF in the UCSC Genome Browser (https://genome.ucsc.edu/). **b** USP2-AS1 is located in HCC cytoplasm predicted by lncATLAS (https://lncatlas.crg.eu/), H19 is mainly located in cytoplasm as a positive contrast, MALAT1 is located in cell nucleus as a negavive contrast. **c** USP2-AS1 almost entirely located in HCC cytoplasm by FISH assay (two cases of HCC tissues, HCC cell lines MHCC97H and Huh7 were used). **d** Relative expression of USP2-AS1 and its related prognosis in TCGA-LIHC.

Fig.S2. The possible diagnostic value of USP2-AS1. **a-b** USP2-AS1 expression of several HCC cells relative to normal hepatocyte cell line LO2 under hypoxia and normoxia by RT-qPCR. **c** USP2-AS1expression of several HCC cells under hypoxia higher than normoxia by RT-qPCR, two-tailed Student's t-test, *$p<0.05$, **$p<0.01$, and ***$p<0.001$, ns means no statistically significant differences. **d** The area under the ROC curve (AUC-ROC) of USP2-AS1 was 0.824. **e** The binding site of USP2-AS1 to YBX1 was predicted through the online website catRAPID express (http://service.tartaglialab.com/update_submission/380192/4af202c73a), the red shaded sections located at 500-1000nt.

Fig.S3. Related probes for the RNA pulldown assays. **a** The length identification of negative control probe Laz, full lengthprobe of USP2-AS1. **b** these quencing identification of negative control probe Laz, full length and truncated probe (A-F) of USP2-AS1. **c** The length identification of truncated probe (A-F) of USP2-AS1.

Fig.S4. The regulatory effects of lncRNA USP2-AS1 on YBX1 and HIF1A proteins. **a** USP2-AS1 changes HIF1α protein levels, as measured by WB. **b** The expression of YBX1 and HIF1α showed a significant positive correlation in the TCGA-LIHC. **c** The positive correlation of expression between YBX1and USP2-AS1in TCGA-LIHC. **d** USP2-AS1 overexpression or knockdown does not affect YBX1 proteins compared with the controls.

Fig.S5. USP2-AS1 promotes HCC proliferation under hypoxia in vitro which can be blocked by YBX1 knockdown. **a-b** EdU cell proliferation assays confirmed USP2-AS1 under hypoxia affected the proliferative capacity of hepatoma cell lines Huh7 and MHCC97H in vitro and can be blocked by YBX1 knockdown, ns means no statistically significant differences.

Fig.S6. USP2-AS1 promotes HCC migration and invasion under hypoxia in vitro and can be blocked by YBX1 knockdown. **a-b** Cell migration and invasion assays confirmed USP2-AS1 under hypoxia affected the migration and invasion capacity of hepatoma cell lines Huh7 and MHCC97H in vitro and can be blocked by YBX1 knockdown. ns means no statistically significant differences.

Table S1 Mass spectrometry proteins from USP2-AS1 RNA pull down in Huh7 cells

Mass spectrometry proteins from USP2-AS1 RNA pull down			
**Proteins**	**Huh7(hypoxia)**		
	**prot_score**	**prot_mass**	**prot_cover**
YBX1	268	35903	29.6
TPM3	261	32987	21.4
TPM1	231	32746	18
TPM2	224	32945	18.7
P4HB	153	57480	5.5
ACIN1	153	152170	3.8
TUBB4B	137	50255	8.8
KRT6B	116	60315	6.7
FIP1L1	114	66601	5.4
NACA	108	205979	0.7
TCOF1	96	152243	1
SAP30BP	92	33964	9.1
HSP90AB1	92	83554	3.7
LUC7L	90	44100	4
CDK11B	89	92734	2.9
MYO1C	88	122461	2.7
YWHAZ	87	27899	5.7
CLINT1	84	68273	4.3

SPTAN1	83	285163	0.4
SF3B1	81	146479	1.1
SNRPF	79	9776	15.1
TUBB2B	78	50377	6.1
RPS15	76	17029	9
OSBPL3	75	102130	1.6
CAVIN1	73	43450	7.2
DYNC1I2	71	71811	3.3
CLTC	68	193260	0.7
CPSF4	67	31261	6.7
RPS27A	66	18296	18.6
LTV1	64	55049	3.2
GPATCH8	62	165010	1.1
HNRNPM	60	77749	3
ICAM1	59	58587	2.6
AP3D1	58	131159	1.1
GNAS	57	111697	1.5
TMOD3	54	39741	2.8
RNPS1	53	34188	4.9
PHGDH	50	57356	2.8
ATP5F1B	49	56525	3
HNRNPUL2	42	85622	3.6

SPTBN1	42	275237	0.5
WTAP	41	44388	2.8
PLD1	40	124790	1.3
MCM7	39	81884	1.7
DDX5	38	69618	1.3
CALU	38	37198	2.5
WDR83	37	35119	4.4
SSB	36	46979	2.7
HSPA1A	34	70294	2
CXXC1	34	77545	1.7
MYO1B	32	132928	0.8
EPRS1	31	172080	0.9
SRSF4	31	56759	1.8
RPL6	31	32765	3.1
HNRNPK	30	51230	1.9
ACTN4	30	105245	1.2
ABLIM1	30	89513	2.2
CP	29	122983	0.9
RPS6	29	28834	3.2
PNN	28	81693	1.3
CROCC	27	228787	0.4
MDH1	26	36631	4.8

PTCHD3	26	87842	1.3
RPL10A	25	24987	3.7
TENM3	23	305093	0.8
CHERP	23	104150	1.4
ZNF215	23	60908	2.7
RNF17	23	187205	0.6
CAPZA1	22	33073	5.2
SPAG8	22	51791	2.5
DYDC2	21	20801	6.8
RASL11A	18	27160	3.3
TRIP12	16	222234	0.6
FGD5	13	161730	0.8

TableS2. Sequences of primers, siRNAs and shRNA used in this study.

qRT-PCR primers		Sequences
USP2-AS1	Forward	GGGAGAGGCAGAGAGATCCA
	Reverse	CAAGGGAGCAGGACAGTGAG
GAPDH	Forward	GGAGCGAGATCCCTCCAAAAT
	Reverse	GGCTGTTGTCATACTTCTCATGG
YBX1	Forward	GGATGGCAATGAAGAGGACAAA
	Reverse	CGTCTGCGTCGGTAATTGAAGT
HIF1A	Forward	TGATGACCAGCAACTTGAGG
	Reverse	CTGGGGCATGGTAAAAGAAA
HIF2A	Forward	GTGCATCATGTGTGTCAACTACG
	Reverse	GGGCTTGAACAGGGATTCAGTC
MAPK1	Forward	GCTTTTCACTTGCGTGCTCA
	Reverse	GGTGCTGTTCCGTGTCACTA
PKM2	Forward	AGAAAGGTGCCGACTTCCTG
	Reverse	CAGCCTCACGAGCTATCAGG
ANKRD37	Forward	ATTCTTACCTGTCGGGGTGC
	Reverse	CTGAGCACGGCAACACAATC
SLC16A3	Forward	CCATGCTCTACGGGACAGG
	Reverse	GCTTGCTGAAGTAGCGGTT
EGR1	Forward	CCCCGACTACCTGTTTCCAC
	Reverse	TTCTGCCGCAAGTGGATCTT
ABCB1	Forward	TTGCTGCTTACATTCAGGTTTCA
	Reverse	AGCCTATCTCCTGTCGCATTA
GLUT1	Forward	GGACTCCATCATGGGCAACA
	Reverse	CTAGCGCGATGGTCATGAGT
LGALS1	Forward	TCGCCAGCAACCTGAATCTC
	Reverse	GCACGAAGCTCTTAGCGTCA
VEGF	Forward	GAGGAGCAGTTACGGTCTGTG
	Reverse	TCCTTTCCTTAGCTGACACTTGT
ShUSP2-AS1	Forward	GGAACTCACAACACACGGGA
	Reverse	TTGCACAAGATGACAGGGCT
YBX1-siRNA1	GGACGGCAATGAAGAAGA	
YBX1-siRNA2	CCACGCAATTACCAGCAAA	
YBX1-siRNA3	GCAGACCGTAACCATTATA	
